# Supplementary material for: The TLR7/8 agonist INI-4001 enhances the immunogenicity of a Powassan virus-like-particle vaccine
Source: bioRxiv. 2024 Dec 3:2024.11.28.625832. Preprint. [Version 1] doi: 10.1101/2024.11.28.625832 (PMC11642962; doi:10.1101/2024.11.28.625832)
Supplement: 1 [file NIHPP2024.11.28.625832V1-supplement-1.pdf]

bioRxiv preprint doi: https://doi.org/10.1101/2024.11.28.625832; this version posted December 3, 2024. The copyright holder for this preprint (which was not certified by peer review) is the author/funder, who has granted bioRxiv a license to display the preprint in perpetuity. It is made available under aCC-BY 4.0 International license.

Supporting information

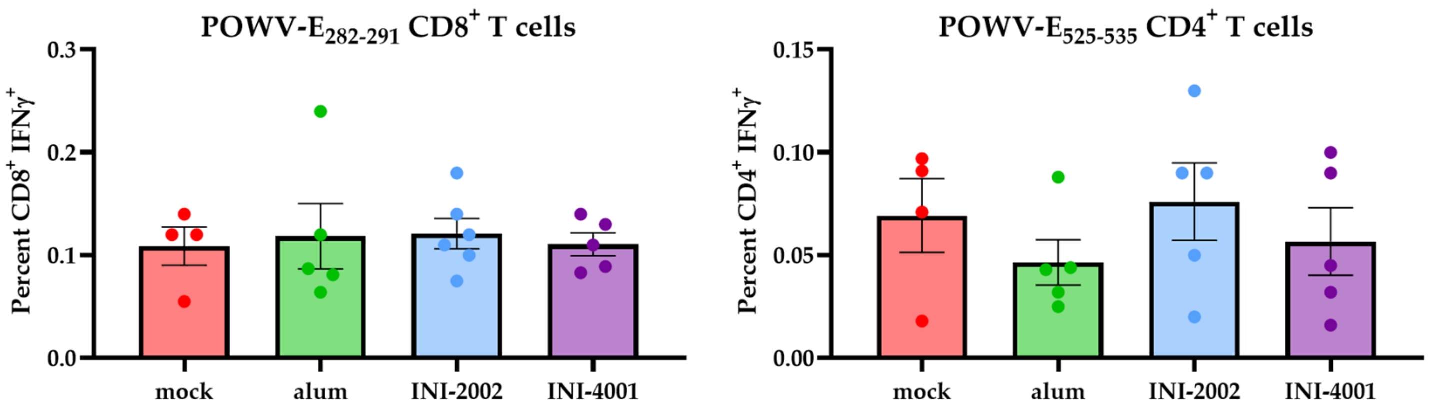

S1 Fig. Vaccination does not induce measurable $CD8^+$ or $CD4^+$ T cell responses. (A-B) Mice prime-boost vaccinated 4 weeks apart *sc* with 10^6 FFUe of VLP alone or adjuvanted with 300μg alum, 1nmol INI-2002, or 10nmol INI-4001. Mock group injected with PBS vehicle alone. n=4-5. Spleens collected 4 weeks post-boost, and splenocytes incubated with POWV-$E_{282-291}$ (A) or POWV-$E_{525-535}$ (B) for $CD8^+$ and $CD4^+$ T cells, respectively. Cells were then stained and gated on CD19- CD4+/CD8+ and intracellularly stained for IFNγ. Data are reported as percentage of $IFN\gamma^+$ T cells either within the $CD8^+$ (A) or $CD4^+$ (B) compartments ± SEM. Data represent one experiment. Statistical significance determined by one-way ANOVA and Šídák's multiple comparisons test to compare treatments to mock; no statistically significant differences were found.
